# Endocannabinoid System: the Direct and Indirect Involvement in the Memory and Learning Processes—a Short Review

**DOI:** 10.1007/s12035-016-0313-5

**Published:** 2016-12-06

**Authors:** Marta Kruk-Slomka, Agnieszka Dzik, Barbara Budzynska, Grazyna Biala

**Affiliations:** Department of Pharmacology and Pharmacodynamics, Medical University of Lublin, Chodzki 4a Street, 20-093 Lublin, Poland

**Keywords:** Endocannabinoid system, Cannabinoid receptors, Memory and learning, Cognition, Animal models of memory

## Abstract

The endocannabinoid system via cannabinoid (CB: CB1 and CB2) receptors and their endogenous ligands is directly and indirectly involved in many physiological functions, especially in memory and learning processes. Extensive studies reported that this system strictly modulates cognition-related processes evaluated in various animal models. However, the effects of cannabinoids on the cognition have been contradictory. The cannabinoid compounds were able to both impair or improve different phases of memory processes through direct (receptor related) or indirect (non-receptor related) mechanism. The memory-related effects induced by the cannabinoids can be depended on the kind of cannabinoid compound used, dosage, and route of administration as well as on the memory task chosen. Therefore, the objectives of this paper are to review and summarize the results describing the role of endocannabinoid system in cognition, including various stages of memory.

## Pharmacology of the Endocannabinoid System

The endocannabinoid system (ECS) is a lipid signaling system, which is functionally active since the early stages of brain development and remains active during both prenatal and post-natal life [1–3]. This system consists of the cannabinoid (CB) receptors, their endogenous ligands, the enzymes for the synthesis and degradation of endocannabinoids, and the reuptake transport system [4].

The discovery of specific CB receptors, followed by identification of their endogenous ligands, gave an opportunity to the extensive research on the significance of this system for the proper functioning of the organism. CB receptors were discovered in late 1980s and then were divided into two different subtypes of G protein-coupled receptors [5]. Currently, two types of CB receptors are known. The pharmacological effects are mainly exerted through the activation of Gi/o protein-coupled membrane receptors CB1 and CB2. Despite the fact that both CB1 and CB2 receptors belong to the group of G protein-coupled receptors and are characterized by significant homology, they differ in their function and specificity of cellular expression [6].

CB1 receptors are located mainly in the central nervous system (CNS), and they are one of the most abundantly expressed neuronal receptors in the CNS, which suggests their important role in the function of the CNS. These receptors are widely expressed in multiple brain areas with the highest concentration in the regions associated with cognition and movement like amygdala, hippocampus, septum, brain cortex, globus pallidus, substantia nigra, cerebellum, and lateral caudate putamen [4]. Additionally, they are also present at lower concentration in a variety of peripheral tissues, both on sensory nerve fibers and in the autonomic nervous system [6–8]. CB1 receptors are localized presynaptically on glutamatergic and gamma-aminobutyric (GABA) acid axon terminals [9]. In the hippocampus, CB1 receptors are located mainly in GABA-ergic, inhibitory interneurons. They are also present in the hippocampal glutamatergic axon terminals, but their concentration is at least 20 times lower than in the presynaptic areas of this brain structure. Activation of CB1 receptors is connected with inhibition of adenyl cyclase as well as calcium channels and leads to activation of potassium channels; thus, it contributes to short-term depression of neurotransmitter release in corticostriatal GABA-ergic and glutamatergic neurons [5]. CB1 receptors are also present on noradrenergic terminals, and their blockade increases release of norepinephrine in limbic regions [10, 11]. Owing to their localization, CB1 receptors control both cognitive process and emotional behavior, including stress, fear, or anxiety [12–17] by modulating neuronal signaling and synaptic plasticity [18].

**Fig. 1:**
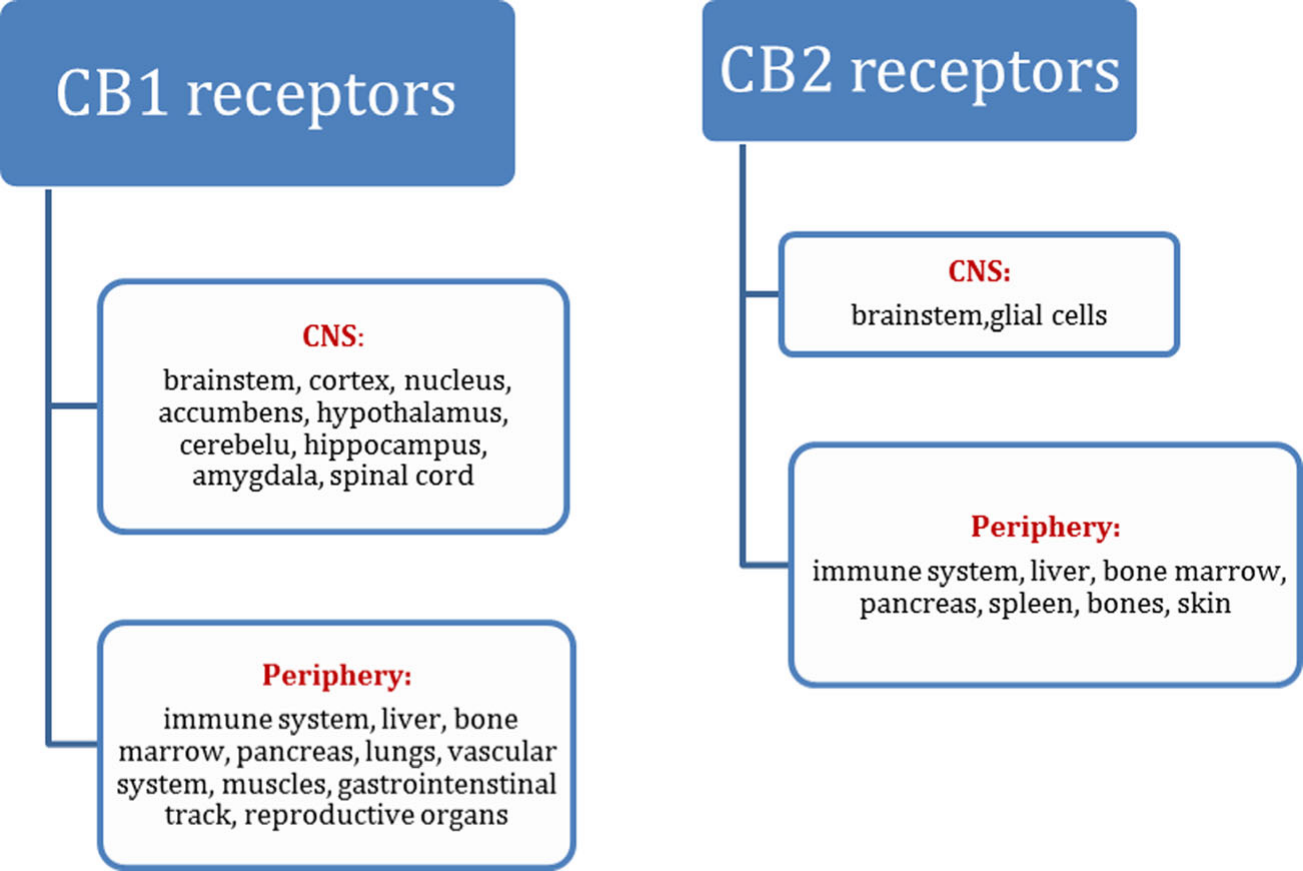
The distribution of CB receptors in the CNS and periphery

In turn, CB2 receptors are present mainly peripherally and are the most abundant in the immune system in a variety of immune cells including B lymphocytes, natural killer cells, monocytes, macrophages, polymorphonuclear neutrophils, and T cells [4, 6]. Thus, they are mainly involved in immune system functions [6, 19]. However, the CB2 receptors have also been found in microglial cells in the CNS. The gathered data suggests that CB2 receptors modulate neuronal function and play a role in psychiatric disorders. Polymorphism of CB2 receptor gene encoding CB2 receptors in humans is related to schizophrenia [20, 21], depression [22], and bipolar disorders [23]. Moreover, in CB2-knockout mice, schizophrenia-like symptoms were observed [24]. Additionally, the CB2 receptors modulate both excitatory [25, 26] and inhibitory synaptic transmissions in the hippocampus [27–29]. It has been reported that the activation of CB2 receptors reduces pain [30], impulsive behavior [31], locomotor activity of rodents [22, 32, 33], and vomiting of ferrets [34]. Stimulation of CB2 receptors also decreases the excitability of peripheral sensory neurons [30], cortical pyramidal neurons [35], and dopaminergic neurons in the ventral tegmental area (VTA) [36] (Fig. 1).

As mentioned earlier, endocannabinoids are synthesized on demand from lipid precursors derived from the enzymatic cleavage of cell membrane constituents in response to neuronal membrane depolarization or immune cell activation and are released from post-synaptic membranes as retrograde messengers onto presynaptic terminals of excitatory or inhibitory character, thus suppressing both inhibitory and excitatory signaling within specific neuronal area. Endocannabinoids control synaptic plasticity by an influence on neurotransmitter release [5, 6, 18]. They have affinity for both CB1 and CB2 receptors [6]. Henceforth, two endogenous cannabinoids (endocannabinoids) were discovered: arachidonoylethanolamide (anandamide (AEA)) and 2-arachidonoylglycerol (2-AG) [5]. They remain the two most studied endogenous substances from the others known so far, including virodhamine, noladin ether, palmitoylethanolamide (PEA), *N*-arachidonoyl dopamine (NADA), *N*-arachidonylglycine (NAGly), oleamide, and oleoylethanolamine (OEA) [37] (Table 1).

**Table 1:**
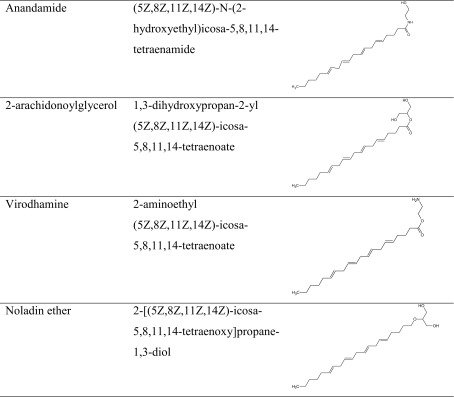

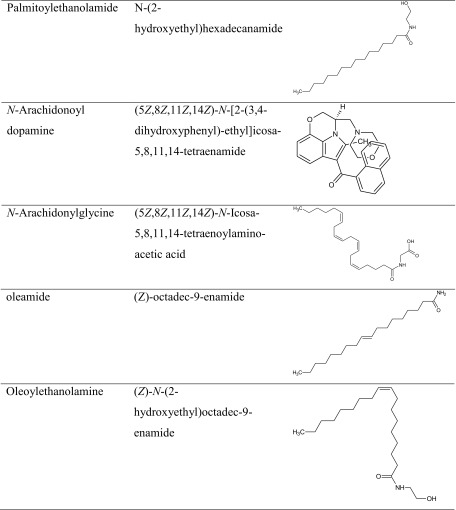
The chemical structure of endocannabinoids

2-AG is mainly produced in the CNS, and AEA is produced at low levels in the periphery and the CNS [38]. Production of endogenous cannabinoids is increased in response to pathogenic stimulus. Particularly important to immune modulation is a fact that the production of endocannabinoids is stimulated by activation of immune cells (macrophages) and dendritic cells, and stimulated immune cells have reduced the expression of endocannabinoid-degrading enzymes [39]. Endocannabinoids are metabolized by degradative enzymes like fatty acid amid hydrolase (FAAH), which metabolizes AEA as well as 2-AG, and monoacylglycerol lipase (MAGL), which metabolizes 2-AG [8].

It should be also noted that there are two novel G protein-coupled orphan receptors GPR55 and GPR119, which have been recently defined as CB receptors [40]. Though showing virtually no apparent homology to either of the classical CB receptors, GPR55 was identified as a novel CB receptor [41]. The CB-sensitive receptor GPR55 was identified and cloned by Sawzdargo et al. [42]. Its presence in the brain, including the hippocampus, has been proved by using quantitative polymerase chain reaction (PCR) [43, 44]. GPR55 activity can be modulated by phytocannabinoids and endocannabinoids [38, 44]. The endocannabinoids that have affinity for GPR55 receptors probably include AEA, 2-AG, PEA, and others [45]. Moreover, recent studies suggest that l-α-lysophosphatidylinositol, which activates GPR55 but not CB1 or CB2 receptors, could also be its endogenous ligand [46, 47]. Contrariwise, cannabidiol (CBD), a major constituent of *Cannabis sativa*, is a GPR55 antagonist, with low affinity for CB1 receptors [44, 48]. In turn, GPR119 receptors are expressed on enteroendocrine L cells of the gastrointestinal tract. They regulate the release of the anti-diabetic peptide glucagon-like peptide-1 [49–51]. These receptors are also found on pancreatic β cells in the islets of Langerhans. OEA is one of the most potent ligands for these receptors, but they are not activated by AEA and only weakly by PEA [41]. However, the pharmacology of both GPR55 and GPR119 is enigmatic, and its adaptive role in the brain remains unknown. Therefore, the explanation of their exact role in the ECS requires further studies.

As we described previously, the ECS, through CB receptors, and its interactions with a multitude of neurotransmitters and receptors are directly and indirectly involved in many physiological and physical functions [52–61]. In the recent years, a large number of studies focused on learning and memory processes. The substances exerting their action through ECS are able to both impair and enhance different phases of memory formation through direct and indirect mechanisms. However, the results of multiple studies show that manipulations performed on the ECS in reference to learning and memory bring contradictory results. Thus, the purpose of this paper is to review and summarize findings connected with the involvement of the ECS in the different memory stages.

## The Role of CB Receptor in the Memory-Related Responses in Animal Models of Memory: CB Receptor Agents

### CB1 Receptor Ligands

The influence of the CB1 receptor ligands on memory and learning processes has been widely documented by various experiments and clinical studies [8, 62–66]. Nevertheless, the results are still contradictory. In this part of the present elaboration, we will summarize the effects of the CB1 receptor ligands, including CB1 receptor agonist and CB1 antagonists (and inverse agonists) on different memory stages.

Studies have demonstrated that an acute administration of synthetic CB1 receptor agonists: CP55940 and HU-210 attenuated acquisition of memory in various animal models, e.g., the water maze test (WMT), the object recognition task (ORT), and the contextual fear conditioning (CFC) test [67–71]. Similarly, Mazzola et al. [68] and others [71] confirmed these effects of direct activation of natural CB1 receptor agonist—Δ9-tetrahydrokannabinol (Δ9-THC). Δ9-THC (3.0, 5.0, and 6.0 mg/kg) injected intra-peritoneally (i.p.) 30 min before the learning trial significantly impaired memory acquisition using the passive avoidance (PA) task in rats. This deterioration was reversed by pretreatment with 1 mg/kg of rimonabant (SR141716A), a CB1 receptor antagonist. Moreover, indirect stimulation of CB1 receptors impaired acquisition of memory in the recognition memory test [72].

In turn, Pamplona and Takahashi investigated whether CB1/CB2 receptor agonist, WIN 55,212-2, is able to influence the acquisition of fear conditioning using tone and contextual versions [69]. They revealed that this compound (2.5 and 5.0 mg/kg, i.p.), administered before conditioning and before testing, impaired memory processes in the CFC and did not affect the freezing behavior induced by tone presentation; therefore, non-state-dependent effects of WIN 55,212-2 on tested animals were observed. During the course of further studies, selective CB1 antagonists (SR141716A and SR147778) were administered, in order to establish whether impaired contextual conditioning would be prevented. Preadministration of SR141716A (1.0 mg/kg, i.p.) or SR147778 (1.0 mg/kg, i.p.) has in fact prevented the impairment. These findings demonstrate that an acute administration of WIN 55,212-2 dose-dependently impairs the acquisition of memory in the CFC test, which is a hippocampus-dependent learning and memory task and does not affect tone fear conditioning, which is considered independent of the hippocampus [73, 74]. Likewise, chronic administration of WIN 55,212-2 significantly impaired spatial memory in rats evaluated in the WMT [75]. Additionally, Kruk-Slomka et al. [64] revealed that WIN 55,212-2 impaired both acquisition and consolidation of memory in PA test in mice. The evidence gathered from this experiment reaffirms that the effects of the CB receptor agonists are selective for the hippocampus-dependent aversive memories in rats.

Contradictory data concerning the influence of CB1 on memory consolidation has also been reported. It has been demonstrated that post-training administration of CB1 receptor agonist HU-210 as well as a combined CB1/CB2 receptor agonist WIN 55,212-2 attenuated consolidation of memory in the CFC test, the WMT, and the ORT [7, 76–78]. Indeed, Maćkowiak et al. [77] investigated the role of CB1 receptors in hippocampal-dependent memory consolidation using HU-210. The results indicated that HU-210 impaired the consolidation of fear memory in the CFC test. These detrimental effects were abolished by a CB1 receptor antagonist AM251. These findings may suggest the involvement of CB1 receptors in memory and learning processes. The results of the studies indicated also that AM251 blocked the effects of HU-210 on freezing behavior but did not affect memory consolidation in the CFC on its own. Thus, the blockade of CB1 receptors does not affect consolidation of contextual memory [79] and disrupts memory consolidation in a step-down inhibitory avoidance (IA) [80, 81].

Similar effects of WIN 55,212-2 on memory consolidation were observed in spatial memory formation using the WMT [78]. Yim et al. administered WIN 55,212-2 systemically and intra-cranially to assess both methods of drug delivery. They demonstrated that this CB1/CB2 receptor ligand impairs the consolidation of long-term spatial memory. Similar long-term memory impairments were observed in both systemic injections and intra-hippocampal infusions of WIN 55,212-2. As CB2 receptors are not expressed in the hippocampus, therefore, the observed impairments provide an indirect support that this effect was reached by targeting CB1 receptors [78]. Nonetheless, it has been noted that intra-basolateral amygdala (intra-BLA infusion) of WIN 55,212-2 facilitated memory consolidation in rats evaluated in the IA task or had no effect in the mentioned animal model [82, 83]. On the other hand, post-training intra-hippocampal injection of this drug impaired consolidation of memory in several behavioral tasks [84]. WIN 55,212-2 was also evaluated in the experiments conducted by Clarke et al. [76]. They examined the effects of post-training activation of hippocampal CB receptors on the consolidation of object recognition memory. The results of the study were in agreement with the evidence provided by Yim et al. [78]. WIN 55,212-2 impaired the consolidation phase of memory formation. Amnestic effect of this compound was completely reversed by coadministration of CB1 receptor antagonist, AM251, as well as mimicked by CB1 receptor agonist, ACEA, but not by CB2 receptor agonist, JWH-015 [78]. These findings are also in agreement with the results published by Moshfegh et al. [85]. They used a step-down PA task as a model of learning. The results indicated that post-training administration of WIN 55,212-2 produced an amnestic response. All the effects described previously endorsed the hypothesis that the memory impairments were due to activation of hippocampal CB1 receptors.

Systemic administration of CB1 receptor antagonists, e.g., rimonabant (SR141716A) or AM251, has been tested in various learning paradigms alone or coadministered with CB1 agonists [61, 63, 64, 81, 86–89].

SR141716A is a selective and potent CB1 receptor antagonist [90]. It also presents features of an inverse agonist [91]. An acute, pretraining administration of SR141716A facilitated the acquisition of memory in rodents observed in the PA test, the elevated T-maze (ETM) test, and social recognition memory task [61, 89] and impaired the acquisition of memory assessed in the spatial memory test [92]. Additionally, systemic, post-training administration of rimonabant enhanced memory consolidation in the radial arm maze (RAM) [89]. Also, Robinson et al. studied the effects of SR141716A on spatial learning and memory formation using the WMT [92]. Two experiments were performed. In the first one, rimonabant was administered i.p., before or immediately after training. The results indicated that systemic administration before training induced deficits in acquisition of spatial reference memory; however, pretraining before drug treatment eliminated this effect. The experiment revealed that rimonabant-induced memory deficits appeared as a result of anxiogenic effects of the drug. Post-training injections had no effect. In the second experiment, rimonabant was administered intra-hippocampally during the training and the results indicated that this drug enhanced acquisition learning and exerted no effect on consolidation of memory. Subcutaneous injections did not affect memory in any way [92].

The results from these described experiments demonstrated that rimonabant produced various effects dependent upon the route of administration, the timing of infusion, and the dose of the drug. Similarly, Lichtman reported an improvement of memory acquisition induced by administration of SR141716A [93]. Likewise, Wolff and Leander showed the enhancement of the consolidation processes when the animals were tested in the RAM test [89]. Furthermore, Wolff and Leander [89] proved a dose of 1.0 mg/kg of SR141716A to be effective. The higher dose of 3.0 mg/kg did not improve the consolidation, what is consistent with the results obtained by Lichtman [93] in the same task. Pro-cognitive effects of rimonabant were also shown in the experiments performed by Takahashi et al. [88]. Administration of 1.0 mg/kg of rimonabant produced an improvement in memory acquisition and consolidation in the ETM task. Neither lower (0.5 mg/kg) nor higher (2.0 mg/kg) doses were able to improve acquisition. Additionally, facilitation of short-term olfactory memory in the social recognition test was described by Terranova et al. [88]. On the other hand, Marsicano and colleagues failed to prove any effect of rimonabant on the consolidation of aversive memories [94]. This result may be accounted to the different mouse strain. The single dose used by Marsicano et al. [94] was also higher than the maximum dose used in the studies described previously.

AM251 is a member of the same CB group of diarylopyrazoles as SR141716A, presenting the features of CB1 receptor antagonist and inverse agonist [4]. The post-training administration of AM251 interfered consolidation of memory-related processes in the step-through IA task or CFC task [82, 87]. De Oliveira Alvares et al. [81] investigated the effects of intra-hippocampal administration of AM251 in two behavioral tasks: the IA and the open field (OF) habituation task. The results indicated that AM251 exerted a disruptive effect on memory consolidation in the IA test, but not in the OF habituation test. Similarly, Kruk-Slomka and Biala [63] confirmed that an acute injection of AM251 improved the short-term and long-term memory stages (acquisition, consolidation, and/or retrieval) in the IA task. The effect seemed to be purely mnemonic since the drug showed no motor performance effects, which could favor a false positive for the intermediate dose in the IA test session. It needs to be highlighted that the amnestic effect took place with the lower, more selective dose, not with the higher one (that one could bind to the non-specific targets in the hippocampus). The different responses observed in two behavioral tasks require explanation. The findings of the study suggest that hippocampal endocannabinoids are not acting upon the consolidation of the OF habituation task. The fact that the OF was not recruited and the IA was sensitive to AM251 raises the possibility that this system requires some degree of aversiveness or alertness to be recruited. The impairing effect of AM251 on memory consolidation was also confirmed by Bucherelli et al. in 2006 [87]. De Oliveira Alvares and colleagues replicated the previous findings [80]. Afterwards, Bialuk and Winnicka [4] performed a study in an attempt to determine the influence of different doses of AM251 on recognition memory. In order to evaluate the effects of AM251 on acquisition of information, the drug was given 15 min before learning trial, and to establish its influence on consolidation of information, it was injected immediately after the trial in the ORT. The results of the study indicated that AM251 significantly improved both acquisition and consolidation of information; however, these effects were observed only when dose of 1 mg/kg was administered. Higher doses did not exert any influence on it. The memory-improving effect is in an agreement with the results obtained in experiments with SR141716A [4].

Interesting experiments in the context of our paper seem to be the studies of Tan et al. [95]. The authors used CB antagonist, agonist, and reuptake inhibitor, AM251, WIN 55,212-2, and AM404, respectively. The substances were administered bilaterally as an intra-BLA and intra-prelimbic (intra-PLC) microinfusion in rats. The results indicated that pharmacological inhibition of intra-BLA CB1 receptor transmission dose-dependently blocked the acquisition of olfactory fear memory, simultaneously leaving unaffected recall and consolidation of these memories in an olfactory fear conditioning procedure. In addition, activation of CB1 receptor transmission or inhibition of the endocannabinoid reuptake within BLA strongly potentiated the acquisition of fear memory. Moreover, fear memory formation, mediated by CB1 receptor, was blocked when the medial prefrontal cortex (mPFC) was pharmacologically inactivated before intra-BLA activation of CB1. These findings are consistent with the report presented by Campolongo et al., which showed that intra-BLA activation of CB1 receptors can potentiate the encoding of associative memory for IA learning [82]. Previous studies conducted by Roche et al. [96] indicated that intra-BLA blockade of CB1 receptor transmission with rimonabant inhibits the formation of context-dependent fear memory. Overall, the previously mentioned findings suggest that CB1 receptor-dependent transmission within the BLA can influence the magnitude of emotional memory encoding. Additionally, the memory-improving effects of AM251 observed in this study were in agreement with the results obtained by Riedel and Davies [97]. They have reported that while the CB1 receptor agonists impair memory formation, the CB1 receptor antagonists reverse these deficits or act as memory enhancers.

Lin et al. studied the effects of another CB1 receptor antagonist AM281 on the formation of contextual fear memory in adult mice [98]. AM281 (2.5 mg/kg) was injected both intra-peritoneally and intra-hippocampally to assess the influence on memory acquisition. These results indicate that CB1 receptor-mediated signaling within the area of hippocampus negatively regulates the acquisition in contextual fear memory task. Thus, AM281 seems to be a good candidate for memory enhancement; however, further studies in animal models of cognitive dysfunctions are still required. In the context of this subject, it should be noted that Wise et al. decided to determine the effects of a relatively novel and potent CB1 receptor antagonist CE on memory processes. CE is structurally distinct from rimonabant. Wise and colleagues observed that CE significantly enhanced memory consolidation in the RAM procedure [99].

As we described previously, the influence of the CB1 receptor ligands on memory and learning processes has been widely documented by various experiments and clinical studies. Although CB1 receptor ligands are able to improve as well as to impair memory, each of them affects memory in a different way. Such contradictory findings may be connected with differences in behavioral tasks used, handling procedures, e.g., time of drug administration, the kind of drug treatment, or other experimental conditions, as well as doses and CB compounds selected. Therefore, future studies may help to discover and describe the precise role and character of different CB1 receptor compounds (Table 2).

**Table 2:**
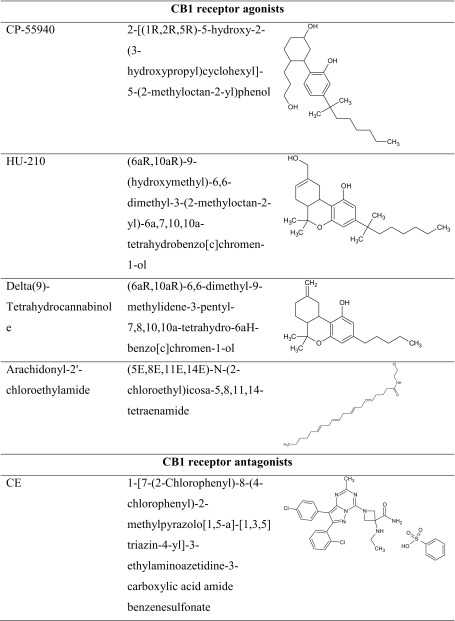

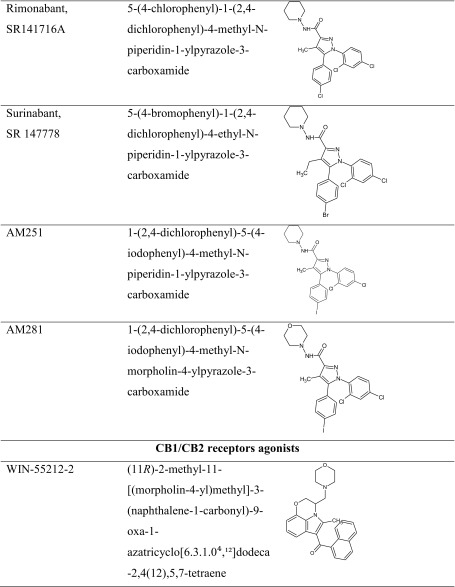
The chemical structure of CB1 receptor ligands

## The Role of CB Receptor in the Memory-Related Responses in Animal Models of Memory: CB2 Receptor Agents

The specific impact of CB2 receptor ligands on the cognition-related processes seems to be more complex and still not precisely explored. In this part of the present elaboration, we can discuss only few results concerning the effects of the CB2 receptor ligands on memory and learning.

The results of the studies suggest that the activation of CB2 receptors evokes diverse effects depending on the brain area. Chronic stimulation of CB2 receptors in the hippocampus increases excitatory synaptic transmission [26], and simultaneously, deletion of CB2 in the same brain structure leads to reduction in dendritic spine density [25]. Chronic activation of CB2 also increases GABA-A receptor expression [28], yet it does not affect the inhibitory synaptic transmission in the hippocampus. CB2 receptor agonists reduce membrane excitability of cortical neurons [35] leaving hippocampal neurons unaffected [26]. CB2 receptor agonists also increase chloride conductance [35].

JWH133 is a selective CB2 receptor agonist. Kruk-Slomka et al. [64] established that the lower dose of JWH133 (0.5 mg/kg) exerted no influence on the acquisition but enhanced the consolidation of long-term memory in the PA test. JWH133 (at higher doses of 1.0 and 2.0 mg/kg) improved the acquisition or consolidation of long-term memory. An acute pretraining and post-training administration improved memory-related responses evaluated in the PA test.

The CB2 antagonist AM630 is one of the most studied exogenous CB receptor ligands. It acts as an inverse agonist both at CB2 and CB1 receptor sites [100]. Kruk-Slomka et al. [64] revealed that AM630 significantly improved memory. The higher doses of AM630 (2.0 and 3.0 mg/kg) induced statistically significant increase in antioxidant properties of brain tissue and evoked long-term memory improvement in behavioral test. However, the lower dose (0.5 mg/kg) was found inactive; it does not alter memory-related responses in the PA test in mice.

It has been revealed in behavioral studies described previously that both a selective CB2 receptor agonist JWH133 and a competitive CB2 receptor antagonist AM630 significantly improved long-term memory acquisition and consolidation in the PA test [64]. In contrast to these findings, García-Gutiérrez et al. [25] have shown that JWH133 enhanced memory consolidation, but AM630 impaired memory-related responses in the step-down IA test.

The enhancement of memory caused by both CB2 antagonist and CB2 agonist obtained by Kruk-Slomka et al. [64] may be connected with pharmacokinetic properties of used CB2 receptor ligands, i.e., JWH133 and AM630. It needs to be underlined that a CB2-selective agent AM630 acts as an inverse agonist rather than as a “silent” antagonist. The inverse efficacy at CB2 receptors and the CB2/CB1 affinity ratio has been indicated for AM630 (CB2/CB1 affinity = 165); therefore, AM630 has been found to act as a low-affinity partial agonist in some experiments but as a low-potency inverse agonist in another study [101]. The pharmacological properties of AM630 are more complex. It has been revealed that AM630 acts as an inverse agonist at CB2 receptors as well as an inverse agonist at CB1 receptors [102]; therefore, it may be proposed that both agonist and antagonist of CB2 receptors used in this study may improve memory and learning processes through CB1 as well as CB2 receptors. Further experiments are required to explain this phenomenon.

To sum up, it should be mentioned that the specific impact of CB2 receptor ligands on the cognition-related processes seems to be more complex and still not precisely evaluated. Similarly as CB1 receptor ligands, CB2 receptor ligands are able to attenuate as well as facilitate memory and learning processes. These different memory effects may be associated mainly with pharmacokinetic properties of tested CB2 receptor ligands as well as with antioxidant properties, exhibited by both agonists and antagonist of these receptors (Table 3).

**Table 3:**
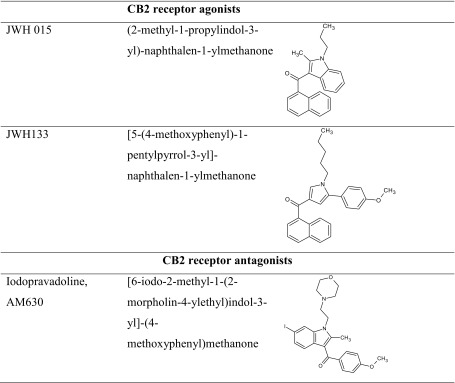
The chemical structure of CB2 receptor ligands

## The Role of CB Receptor in the Memory-Related Responses in Animal Models of Memory: CB Receptor Deficiency

In order to establish the role of CB receptors more accurate and disentangle the role of endocannabinoid system in memory formation, two strands of research have been implemented: knockout mice deficient for CB receptor as well as aforementioned administration of selective CB receptor antagonist.

Litvin et al. used a genetic knockout of CB1 receptors (CB1KOS) in order to evaluate the role of these receptors in memory formation processes [9]. The CB1KOS and the animals that received the CB1 antagonist AM251 showed enhanced levels of social memory relative to their respective controls in a social discrimination test. These results emphasize the role of CB1 receptors in memory formation. The endocannabinoids bind to CB1 receptors in various brain regions to modulate behavioral functions in relation to cognition, emotionality, and stress [12, 103]. These results delineate the effects of CB1 receptor inactivation by utilizing convergent genetic and pharmacological approaches. These paths of experiments produce similar behavioral profiles resulting in enhancing memory acquisition in the social discrimination test with some differences, which can attest to discrepancies between these manipulations. These results extend the role of the ECS in mood and memory [104] and simultaneously are in line with the reports describing a specific role of CB1 receptor in these processes [105]. The results achieved by Litvin et al. [9] of increased cognitive abilities in the CB1KOS mice are consistent with the previous reports describing enhanced cognitive performance in several other tests like active avoidance memory [106], CFC [107], and ORT [108, 109]. It has been also reported that CB1-deficient mice display normal acquisition and impaired extinction of both spatial reference memory [110, 111] and cued fear memory [59]. CB1KOS mice also present reduced working memory [58]. Although the parameters of PA concerning the memory-related effects stayed unaffected [36], the contextual fear memory was reported both to be enhanced and to be impaired [56].

Similarly, for a complete understanding of the mechanism underlying the action of CB2 receptors, it will be necessary to determine the role of CB2 receptors in regulating various properties of synaptic transmission. It also needs to be evaluated whether the affected immune functions in CB2 receptor genetic knockout mice (CB2KOS) influence the processes involved in learning and memory. Li and Kim [112] utilized both CB2-deficient mice and acute blockade of CB2 receptors by AM630. The results indicated that the inhibition of CB2 by a specific CB2 receptor antagonist AM630 had no effect on memory acquisition in contrast to the knockout of CB2 receptors. The findings indicated that acquisition of spatial working memory evaluated in Y-maze in CB2KOS was enhanced in comparison to mice examined in the WMT. The results also indicated that CB2 receptors play diverse roles in regulating memory. Thus, taken together, the results imply that the effects of CB2 receptor blockade (either through genetic deficiency or pharmacological inhibition) are variable. Acute blockade with AM630 exerts no effect on memory acquisition, implying that downregulation of CB2 receptors needs to be prolonged to induce such effects [112].

These all findings indicate that normal acquisition of cued fear memory is common for both CB1- and CB2-deficient mice, but alterations in the working memory are opposite. Overall, conclusion leads to the statement that CB1 and CB2 receptors play a role in modulation of memory processes. Once the role of each type of receptor is fully characterized, either CB1 or CB2 receptor can be selectively targeted for pharmacological therapeutics to induce the desired results and avoid the unwanted ones.

## The Role of Endocannabinoids in the Memory-Related Responses in Animal Models of Memory

As we mentioned previously, AEA and 2-AG are two main endocannabinoids in the CNS. Lin et al. [2011] examined the impact of AM404, an AEA reuptake inhibitor, on the acquisition of memory in mice using the CFC paradigm [98]. AM404 was administered into the dorsal hippocampus 15 min prior to the conditioning session. The outcome of the experiment indicated significant suppression of the fear memory. Moreover, Lin et al. [98] confirmed that the inhibitory effect of AM404 on fear memory formation was mediated by the activation of CB1 receptor. Taken together, they concluded that AEA-mediated activation of CB1 receptor contributes negatively to the acquisition of contextual fear memory.

The level of AEA may be also increased by the usage of FAAH inhibitor. FAAH inhibitor, URB597, increases AEA levels at those neuronal sites and regions of the brain, where AEA is synthesized and released, producing a neuron-specific activation of CB1 receptor in those areas. On the contrary, systemic administration of CB1 receptor agonist such as Δ9-THC produces global activation of all CB1 receptors in the whole brain [68].

Mazzola et al. [68] accomplished the inhibition of FAAH by administering URB597 [68]. The effects of URB597 were studied both alone and after pretreatment with rimonabant in the PA paradigm. The findings of this study revealed that URB597 (0.1–1.0 mg/kg), injected 40 min before the learning trial, had a significant enhancing effect on memory acquisition. Further testing demonstrated that the memory-enhancing effects were inhibited after the pretreatment with 1.0 mg/kg rimonabant. These results are consistent with the previous studies suggesting that FAAH inhibition enhances place memory acquisition in the WMT procedure [113]. The effects of URB597 on acquisition were also studied [114]. In this experiment, the authors evaluated the effects and interaction between URB597 and WIN 55,212-2 using the PA test [114]. Learning and memory impairment was elicited by WIN 55,212-2 (1.0 mg/kg) administered 30 min before the acquisition trial in rats. URB597 (0.1, 0.3, 1.0 mg/kg) or SR141716A (1.0 mg/kg) was injected 10 min before WIN 55,212-2 or URB597, respectively. The results indicated that URB597 at the dose of 0.3 and 1 mg/kg enhanced memory acquisition in the PA test. The dose of 0.1 mg/kg exerted no effects. The cognitive-enhancing effects were blocked by SR141716A. This study also revealed that SR141716A injected separately had no effects on cognition. In conclusion, these findings suggest that URB597 has potential to protect against memory deficits produced by CBs. The results of this study are in accordance with the other studies in which stimulation of the endogenous CB signaling with URB597 enhances acquisition in the PA learning and aversively reinforced spatial memory tasks [68, 86, 113].

As CB1 agonists exert amnestic effects and URB597 increases endogenous level of CB1 agonist AEA, findings that URB597 enhances memory and this improvement can be inhibited by CB1 receptor antagonist are puzzling. It is possible that learning improvement produced by FAAH inhibitor is not actually mediated by CB1 receptor but is blocked by SR141716A due to its inverse agonist effects on CB1 receptor.

De Oliveira et al. investigated the role of AEA upon the different phases of memory processing [37]. The results were evaluated in the step-down IA task. The findings of the study indicated that post-training infusion of anandamide facilitated memory consolidation. It is worth noting that only the small dose of AEA infused into the dorsal hippocampus of the male Wistar rats exerted enhancing effect on memory. Any memory-enhancing effect was observed after the administration of higher doses. The lack of its efficacy at higher doses may be explained by its binding to different areas. At the low dose, AEA may mainly target the CB1 receptors.

It should also be noted that the effects of FAAH inhibitor, OL-135, and of genetic deletion of FAAH in mice were studied [115]. Both of these manipulations enhanced the acquisition of spatial learning in the WMT. This enhancement was inhibited by pretreatment with rimonabant, suggesting the role of CB1 receptors in the observed effects [113, 115, 116].

The research concerning FAAH inhibition opens a new approach for developing medications that act indirectly by enhancing the actions of endogenous lipid amide mediators, where they are synthesized and released. It is worth mentioning that FAAH inhibition might be related to a wide spectrum of therapeutic actions and could also share some of the adverse effects of cannabis; therefore, it is prominent that URB597 possesses potentially beneficial properties and displays no indication of liability for abuse or dependence. Therefore, it has been suggested to improve therapeutic interventions in memory deficit cases (Table 4).

**Table 4:**
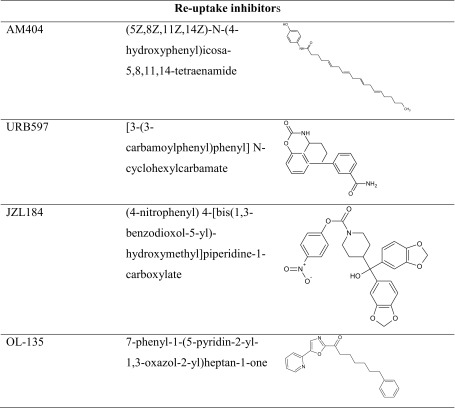
The chemical structure of reuptake inhibitors of endocannabinoids

## Conclusion

The results of the studies described in this elaboration summarize the impact of CBs on different stages of memory formation. Many preclinical studies have evaluated the multidirectional effects of compounds that directly affect the functioning of the ECS (CB receptor ligands), as well as compounds that modulate this function indirectly (inhibitors that degrade endocannabinoids in the brain).

The modulation of the influence of the CB receptor ligands on the different memory stages was widely evaluated in the behavioral studies. Although both CB1 and CB2 receptor ligands are able to improve as well as to impair memory, each of them affects memory in a different way and this subject is still unexplored. Thus, further studies, not only behavioral experiments, but also molecular (e.g., the assessment of the density of the CB receptors in different brain areas: hippocampus, prefrontal cortex) and biochemical (e.g., the influence of CB receptor ligands on the neurotransmitter and metalloproteinase levels in the brain or on the oxidative stress biomarkers) are necessary. The results from these interdisciplinary experiments may provide new information concerning the therapeutically beneficial properties of the ECS in the brain.

## Abbreviations

2-AG: 2-Arachidonoylglycerol
Δ-9-THC: Δ-9-Tetrahydrokannabinol
AEA: Anandamide
Ca ^2+^: Calcium ions
CB: Cannabinoid
CB1KOS: CB1 receptor genetic knockout mice
CB2KOS: CB2 receptor genetic knockout mice
CFC: Contextual fear conditioning
CNS: Central nervous system
ECS: Endocannabinoid system
ETM: Elevated T-maze
FAAH: Fatty acid amid hydrolase
GABA: Gamma-aminobutyric acid
IA: Inhibitory avoidance
Intra-BLA: Intra-basolateral amygdala
Intra-PLC: Intra-prelimbic
i.p.: Intra-peritoneally
MAGL: Monoacylglycerol lipase
NADA: *N*-arachidonoyl dopamine
NAGly: *N*-arachidonylglycine
OEA: Oleoylethanolamine
OF: Open field
ORT: Object recognition task
PA: Passive avoidance
PCR: Polymerase chain reaction
PEA: Palmitoylethanolamide
RAM: Radial arm maze
VTA: Ventral tegmental area
WMT: Water maze test
